# Advancing Breast Cancer Research Through Collaborative Computing: Harnessing Google Colab for Innovation

**DOI:** 10.7759/cureus.57280

**Published:** 2024-03-30

**Authors:** Sydney T Lam, Jonathan W Lam, Akshay J Reddy, Longines Lee, Zeyu Yu, Benjamin E Falkenstein, Victor W Fu, Evan Cheng, Rakesh Patel

**Affiliations:** 1 Medicine, California University of Science and Medicine, Colton, USA; 2 Medicine, California Health Sciences University, Clovis, USA; 3 Internal Medicine, Quillen College of Medicine, East Tennessee State University, Johnson City, USA

**Keywords:** pathology, machine learning, oncology, artificial intelligence, breast cancer

## Abstract

This investigation explores the potential efficacy of machine learning algorithms (MLAs), particularly convolutional neural networks (CNNs), in distinguishing between benign and malignant breast cancer tissue through the analysis of 1000 breast cancer images gathered from Kaggle.com, a domain of publicly accessible data. The dataset was meticulously partitioned into training, validation, and testing sets to facilitate model development and evaluation. Our results reveal promising outcomes, with the developed model achieving notable precision (92%), recall (92%), accuracy (92%), sensitivity (89%), specificity (96%), an F1 score of 0.92, and an area under the curve (AUC) of 0.944. These metrics underscore the model's ability to accurately identify malignant breast cancer images. Because of limitations such as sample size and potential variations in image quality, further research, data collection, and integration of theoretical models in a real-world clinical setting are needed to expand the reliability and generalizability of these MLAs. Nonetheless, this study serves to highlight the potential use of artificial intelligence models as supporting tools for physicians to utilize in breast cancer detection.

## Introduction

Breast cancer, a prevalent and impactful form of malignancy, has garnered increased global attention due to its wide-ranging health implications [1]. Since 2020, female breast cancer has dethroned lung cancer as the leading cause of global cancer incidence with an estimated 2.3 million new cases and about 685000 deaths and is expected to increase by 91% to 4.4 million new cases by 2070 [2,3]. This disease is characterized by the uncontrollable proliferation of cells within the breast tissue, with the potential to metastasize if not detected and managed promptly [4]. Interestingly, breast cancer can develop from various structures within the breast, including the milk ducts and lobules [5]. The current standard of treatment for treating breast cancer includes a plethora of interventions, such as surgery, chemotherapy, endocrine therapy, immunotherapy, radiotherapy, and targeted therapy. Importantly, breast cancer is not limited to women; it can affect individuals of all genders [6]. This complex ailment underscores the need for comprehensive research and targeted interventions to improve diagnosis and treatment outcomes [7]. 

Early detection and diagnosis of breast cancer plays a significant role in reducing breast cancer death rates in the long term, with a five-year survival rate of 97% found in some studies [8]. To achieve that end, various diagnostic techniques are employed in detecting and assessing breast cancer. Mammography, for instance, provides a non-invasive means to visualize internal breast structures and detect abnormalities at early stages [9]. The field of breast cancer diagnosis also encompasses methods like chest X-ray, ultrasound, magnetic resonance imaging (MRI), and biopsy procedures, each contributing to a more accurate understanding of the disease [10]. These diagnostic modalities empower medical professionals to make informed decisions about patient management. Even within the past 50 years, the proliferation of the use of modern technology in clinical applications has made it possible to not only detect breast cancer earlier but also ensure less disfiguring outcomes of primary therapy, where the goal is to completely remove or eliminate all the cancer cells [11]. 

The global rise in breast cancer incidence is being met with advancements in artificial intelligence and deep learning [3,12]. Convolutional neural networks (CNNs), a subset of deep learning algorithms designed for processing images, excel in tasks like image recognition, classification, and analysis [13]. CNNs surpass traditional machine learning in accuracy and precision for breast cancer diagnosis and classification [14]. This paper aims to harness a CNN model to differentiate between benign and malignant breast histopathological images, offering physicians and pathologists a more accurate, efficient diagnosis process and improving breast cancer screening's effectiveness.

## Materials and methods

Methodology

The purpose of the study was to develop a malignant breast cancer detection model through the use of 1000 breast cancer images from a dataset through the Kaggle online platform (citation). The data of 1000 images included 500 benign breast cancer images as well as 500 malignant breast cancer images. Machine learning models were used to identify certain patterns or identification from the images for the model to learn how to identify between the two categorizations. A machine learning model, such as CNNs, was used to capture certain patterns from the breast cancer images. CNN worked as a deep learning model specific to image analysis through specialized filters, which allowed it to learn and extract hierarchical features from input images. This allowed it to recognize certain patterns or structures in the image. The deep learning model worked by using Google Cloud’s collaborative platform (Google LLC) to create a CNN model to correctly identify patterns and unique features that were associated with malignant breast cancer, which allowed the model to distinguish the difference between a benign and malignant tumor. When using the dataset, it was first divided into three separate categories: (i) training, (ii) validation, and (iii) testing. The training and validation categories were used to train the model while the testing category was used to test the model’s efficacy. 80% of the total images were used for training while 10% was used for validation and the remaining 10% were used for testing the model. The breast cancer images were selected from a diverse pool in order to ensure that they were representative of sample benign and malignant breast cancer cases. The dataset was acquired from Kaggle.com, which was a reliable resource. The images derived from Kaggle.com were previously diagnosed by physicians and have been reviewed for accuracy. The division of categories was an essential part of training the model as the largest portion of the images was dedicated to training to ensure the model’s reliability. The testing category provided a sample for a final evaluation in order to assess whether the model was successful in its previous categories. Both benign and malignant images were shown and tested in order to gauge the efficacy of the detection model.

Ethical considerations

Due to the use of publicly accessible data through Kaggle.com, the investigation was exempted from an Institutional Review Board approval. Since the data was publicly accessible, there was full protection of personal information when considering anonymity and confidentiality. 

## Results

The primary objective of this study was to develop a robust system capable of discerning whether an image depicting a breast tumor is malignant or benign. This objective was pursued through the application of machine learning algorithms (MLAs) to a breast cancer image dataset, with Kaggle.com serving as the primary data source. The developed model exhibited a commendable area under the curve (AUC) of 0.944, signifying its potential to accurately classify breast cancer images as either benign or malignant. Further analysis revealed a precision value of 92%, affirming the model's capacity to correctly identify malignant breast cancer images. Additionally, the recall value of 92% underscored the model's effectiveness in recognizing all positive cases, while the sensitivity value of 89% demonstrated its ability to correctly identify true positives, particularly in identifying malignant images. The specificity of the model, representing its rate of correctly identifying benign images, yielded a substantial value of 96%. The accuracy of the model, which gauges its proficiency in correctly identifying malignant and benign images, stood at 92%. In light of these results, it is evident that the model has demonstrated a high degree of accuracy in distinguishing between malignant and benign cases in breast cancer imaging. While further evaluation and refinements may be warranted to optimize the model's performance, the present findings underscore its potential as a valuable tool in the field of breast cancer diagnosis.

## Discussion

Malignant tissue can manifest in various parts of the body, including the breasts, presenting an ongoing challenge for physicians in terms of detection and diagnosis. This study aimed to investigate the potential of MLAs, deep learning, and artificial intelligence in breast cancer detection. Through the utilization of a dataset, we successfully developed a model capable of accurately distinguishing between benign and malignant breast cancer images. This underscores the significant role that machine learning applications can play in the field of medicine, particularly in enhancing the precision and efficacy of malignant breast cancer detection.

Traditionally, pathology relies on H&E staining of breast cancer cells to determine the benign or malignant nature of tumor biopsy [15]. Previous studies integrating MLAs in mammography breast cancer readings improved cancer detection by radiologists compared to unaided readings [16]. We hope to, in a similar vein, provide pathologists with additional data and support, enabling them to enhance accuracy and make well-informed assessments regarding cell pathology, ultimately aiding in distinguishing between benign and malignant conditions. The model that we developed not only aims to achieve this goal but also highlights an additional modality of model training; Google Collaborative's Vertex AI platform is different from platforms utilized in previous studies.

**Figure 1:**
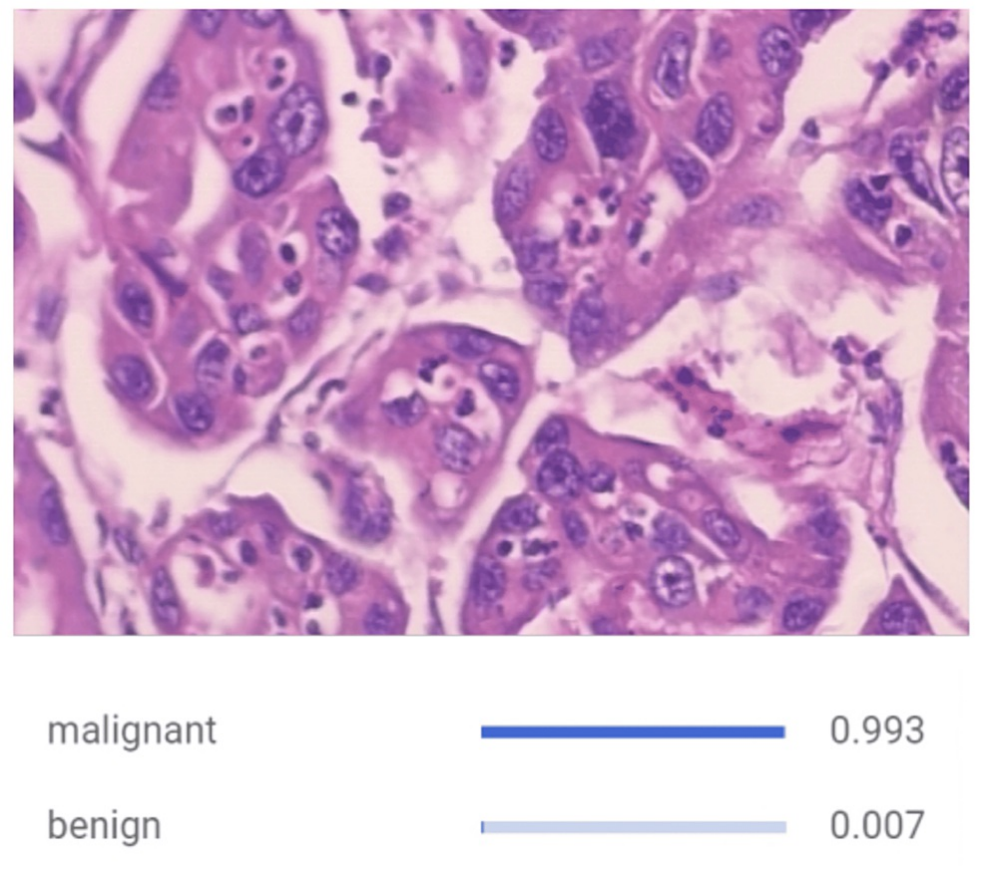
CNN Classifying Malignant Breast Tissue Through Histopathological Image Analysis. The input to the CNN is an image of breast tissue displaying phenotypical characteristics of malignancy, including disorganized cell growth, hyperchromasia, enlarged nuclei, and increased mitotic activity. At the bottom of the figure, the CNN correctly identifies the input image as cancerous. CNN, convolutional neural network

**Figure 2:**
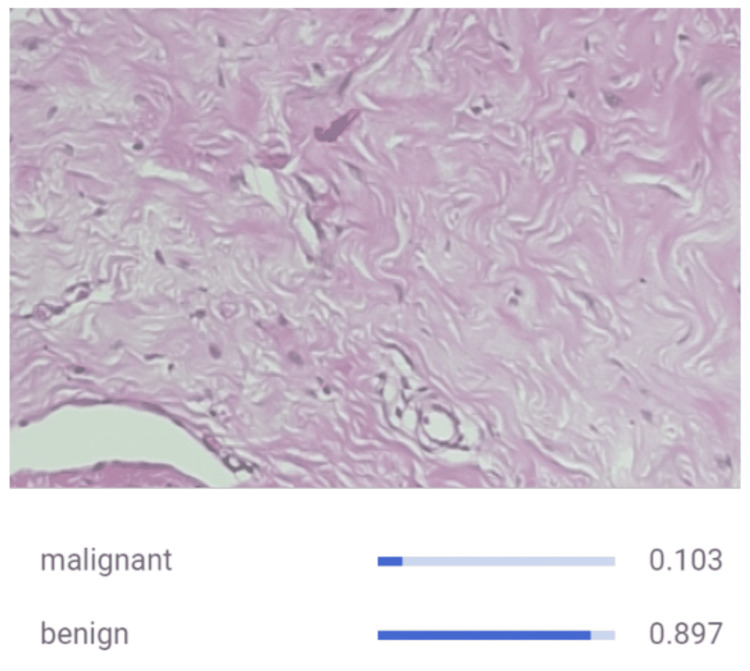
CNN Classifying Benign Breast Tissue Through Histopathological Image Analysis. The input to the CNN is an image of benign breast tissue that is marked by organized cell growth, normal mitotic activity, and normal-sized nuclei. At the bottom of the figure, CNN correctly identifies the slide as benign tissue. CNN, convolutional neural network

**Figure 3:**
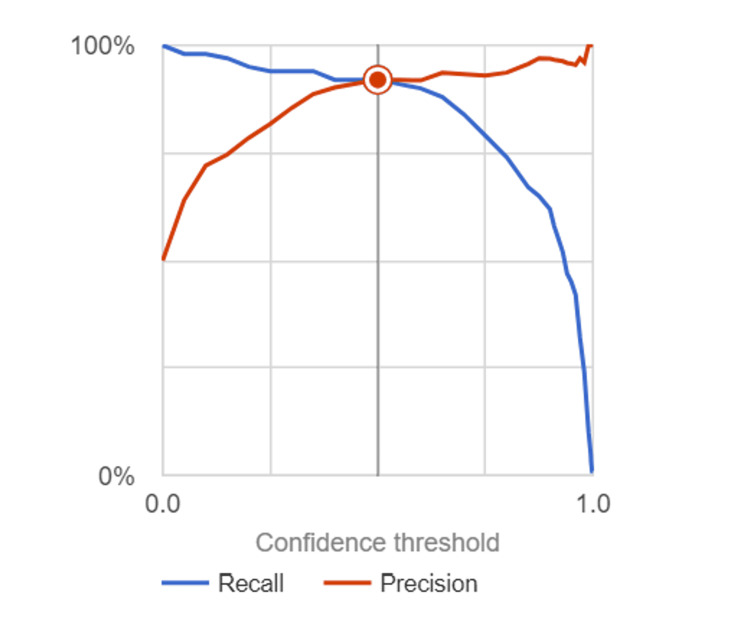
Precision-Recall Curve. The precision-recall curve illustrates the trade-off between precision and recall in classifying breast cancer histopathological images using CNN Model. CNN, convolutional neural network

**Figure 4:**
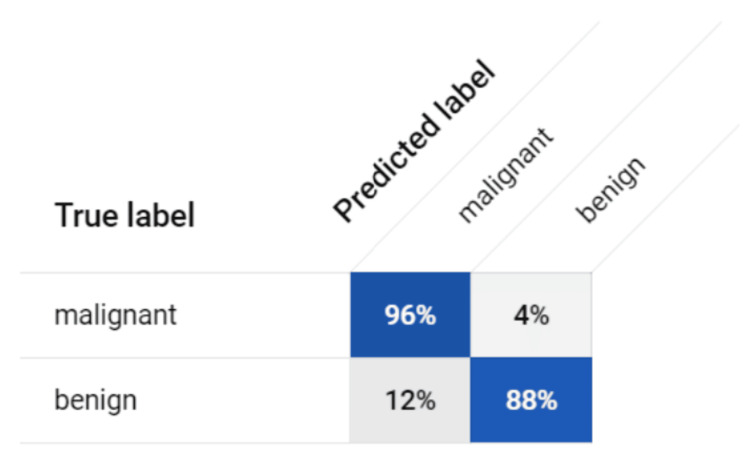
Confusion Matrix. Figure displays confusion matrix values from CNN that were used to calculate accuracy, precision, recall, specificity, and F1 score. CNN, convolutional neural network

Inspired by the biological neural network of the human brain, CNN models help work around morphological classification limitations from traditional algorithms by efficiently learning from image datasets during the training process before accurately classifying them [17]. Our CNN model demonstrated notable proficiency through metrics such as precision, recall, sensitivity, specificity, and accuracy. Its capacity to discern malignancy from benign cells underscores its potential for clinical application, relying on specific patterns and features within the images. The model was successfully able to identify malignant images as seen in Figure 1 as well as correctly identifying benign images in Figure 2. Furthermore, an F1 score of 0.92 was computed, affirming the model's overall performance as seen in Figure 3. This shows that the model is accurate in making the correct prediction. All the values were derived from the information contained within the confusion matrix found in Figure 4. With an F1 score of 0.92 and an AUC of 0.944, the model showed to be accurate in its identification, which shows that it could be successful when implemented in a case. Though other studies have employed CNN models that have demonstrated a higher AUC, it should be noted that those studies also trained their models with a larger sample size of data before deploying their models [14,18,19]. If trained over a larger sample size, our model may also demonstrate an increase in F1 score and AUC. The real-world application of using AI to detect malignant or benign breast cancer cells is feasible as seen by the success of the model.

Future considerations

However, it is important to acknowledge certain limitations within this study. While many images were utilized, the dataset may not encompass an extensive range of breast cancer variations, potentially affecting the model's clinical applicability. Additionally, our model can only generally distinguish between malignant and benign tissue histology. Given this, it would be prudent to develop a model that can help pathologists not only identify whether a tissue sample is malignant or benign but also assist pathologists in differentiating among the different types of breast cancer that are typically diagnosed through histology such as ductal carcinoma in situ, invasive ductal carcinoma, invasive lobular carcinoma, or triple-negative breast cancer. 

Factors including variations in image quality could also have a minor influence. Future studies could consider implementing automated image upscaling and enhancements to investigate possible improvements in AI model efficacy. Incorporating larger and more diverse datasets would bolster the model's effectiveness and reliability.

Furthermore, it is crucial to consider the implications of integrating MLAs in patient care and practicality. The demonstrated potential of MLAs in breast cancer imaging holds great promise for the field of medicine. This study hopes to contribute to the ongoing efforts to refine oncology diagnosis and decision-making, providing a glimpse into the future capabilities of these technologies. The models developed so far have the potential to aid healthcare professionals in diagnosing breast cancers, ultimately enhancing patient care. This research showcases the power of artificial intelligence in crafting a robust detection model through breast cancer imaging. Ongoing research remains imperative to propel technological advancements in oncology.

## Conclusions

Breast cancer continues to be a prevalent form of malignancy and a challenge to our public health systems. Even with new advancements in diagnostic techniques, the global attention to breast cancer and its challenges demands increasingly innovative tools for approaching breast cancer detection. In this study, we evaluated the accuracy of MLAs in differentiating between malignant and benign images of breast cancer tissue in an effort to highlight the potential of using artificial intelligence models as an aid in detecting and assessing breast cancer. Utilizing a standardized statistical model can not only help physicians make better-informed decisions but also provide them with a tool to make more consistent diagnoses. Though further research needs to be done to improve upon the reliability and generalizability of the model, the results of this study have implications for future clinical applications, such as in medically underserved areas where oncologists may not be immediately and readily accessible or in areas with high patient volume and limited medical staffing. With much further refinement and testing, artificial intelligence can be a powerful tool that allows physicians to augment the current arsenal of diagnostic modalities to make better-informed decisions and improve patient outcomes.
